# Whole systems approaches to diet and healthy weight: A scoping review of reviews

**DOI:** 10.1371/journal.pone.0292945

**Published:** 2024-03-13

**Authors:** Gavin Breslin, Olujoke Fakoya, Wendy Wills, Nigel Lloyd, Charis Bontoft, Amander Wellings, Sian Harding, John Jackson, Katherine Barrett, Adam P. Wagner, Lisa Miners, Honey-Anne Greco, Katherine E. Brown

**Affiliations:** 1 School of Psychology, Queen’s University Belfast, Belfast, United Kingdom; 2 Department of Psychology, Sport and Geography, School of Life and Medical Sciences, College Lane, University of Hertfordshire, Hatfield, United Kingdom; 3 Centre for Research in Public Health and Community Care (CRIPACC), School of Health and Social Work, College Lane, University of Hertfordshire, Hatfield, United Kingdom; 4 Member of the PHIRST Public Involvement in Research Group, School of Life and Medical Sciences & School of Health & Social Work, University of Hertfordshire, Hatfield, United Kingdom; 5 Norwich Medical School, University of East Anglia, Norwich Research Park, Norwich, United Kingdom; 6 National Institute for Health Research (NIHR) Applied Research Collaboration (ARC) East of England (EoE), Cambridge, United Kingdom; LSHTM: London School of Hygiene & Tropical Medicine, UNITED KINGDOM

## Abstract

**Background:**

Obesity is a global epidemic affecting all age groups, populations, and income levels across continents, though is known to disproportionately affect socioeconomically disadvantaged populations. The causes of obesity are complex, informed by diet and weight practices, but shaped by social, commercial, and environmental factors and government policy. Consequently, a Whole System Approach (WSA)–which considers the many causes of obesity and shifts the focus away from individuals as points of intervention and puts an emphasis on understanding and improving the system in which people live–is required. This scoping review of reviews aims to: determine how WSAs to diet and healthy weight have been implemented and evaluated nationally and internationally; to determine what models or theories have been used to implement WSAs; describe how WSAs have been evaluated; determine if WSAs are effective; and to identify the contribution of the public and/or service users in the development of WSAs.

**Method:**

Systematic searches were carried out using CINAHL, Scopus, PsycINFO (ProQuest), the Cochrane Library, and MEDLINE. Included review papers were those that focused on the application of a whole system approach to diet and/or healthy weight, and/or reported the theory/model used to implement or simulate this approach. Databases were searched from 1995 to March 2022 using a combination of text and Medical Subject Headings (MeSH terms). In addition, reference sections of identified articles were examined for additional relevant articles. Covidence software was used to screen titles and abstracts from the electronic databases and resolve conflicts.

**Results:**

A total of 20,308 articles were initially retrieved; after duplicate removal 7,690 unique title and abstracts were reviewed, and 110 articles were selected for full text review. On completion of full text review, 8 review articles were included for data extraction. These included: one umbrella review, four systematic reviews, a rapid review, and two literature reviews (one of which was on strategic reports written for government and public health policy). Evaluations of WSA were mainly process evaluations although health outcomes were assessed in some studies. Several conceptual frameworks or mathematical modelling approaches have been applied to WSAs for diet, healthy weight, and obesity to inform their planning or delivery, and to understand/map the associated systems. Common mathematical approaches include agent based or System Dynamic Modelling. Underlying both conceptual and mathematical models is an understanding how the elements of the complex systems impact each other to affect diet, healthy weight, and obesity. WSA implementations have reported some success in positively impacting health outcomes including reducing Body Mass Index, reducing sugary food intake, and increasing physical activity. Public and user involvement in WSA was not widely reported.

**Conclusion:**

The application of WSA to diet and healthy weight shows promise, yet the research is lagging behind their implementation. Further robust evidence for using WSA to address diet and healthy weight are required, including incorporating process and outcome evaluations (perhaps using established approaches such as Systems Dynamic Modelling). Furthermore, the analysis of epidemiological data alongside longitudinal process and outcome evaluation regarding the implementation of a WSA is required.

## Introduction

Excess weight and obesity is a complex problem that affects all ages and socioeconomic groups [1] but disproportionally affects those living in greatest socioeconomic deprivation [2]. It is estimated that globally more than 1.9 billion adults live with overweight, 650 million live with obesity, and 38.2 million children under the age of 5 were living with overweight or obesity in 2019 [3]. Obesity has been linked to a range of comorbidities in adults including diabetes, cardiovascular disease, hypertension, and certain types of cancer [4]. Thus, reductions in the prevalence of obesity would improve the quality of life of individuals by reducing the years lived with illnesses associated with these conditions. Moreover, it would also reduce the mortality rate and enhance the life expectancy of individuals [5].

Globally, poor diet is a leading risk factor for the development of excess weight or obesity [6]. There is a broad agreement, though, that causes of obesity are complicated and embedded in complex systems of interdependent causal factors [7] that operate at multiple levels and across settings [8]. The World Health Organisation’s European Regional Obesity Report 2022 suggested policy interventions that target environmental and commercial determinants of poor diet at a population level are likely to be most effective in reducing obesity, addressing dietary inequalities, and achieving environmentally sustainable food systems [9]. As behaviours that lead to obesity are highly socially patterned, changing them in an equitable fashion requires simultaneous multiple interventions at a range of levels [10]. As there is no one solution to tackling obesity, successful action to promote healthy weight and diet across the life course requires a coordinated collaborative approach [1].

Whole Systems Approaches (WSAs) have become a popular guiding framework for planning public health responses to combating social and health problems with poor diet and unhealthy weight being recently targeted [11–14]. However, a shared definition or model of what a WSA should look like in practice (i.e., how it should be developed and effectively implemented) is lacking. Further, the meaning of the term ‘systems approach’ varies for different authors and organisations, often with contradictory definitions [15], and despite the increased interest in, and attempts to apply WSAs to address overweight and obesity, robust evidence for their effectiveness remains in its infancy [12, 14, 16, 17]. In many cases evaluations have been process focused rather than both process and outcome focused (where changes in obesity-related outcomes are assessed alongside changes to structures and processes in the system). In an evidence review commissioned by the National Institute for Health Care and Excellence (NICE) to identify key elements of a WSA to obesity [15], it was reported that an ‘authentic’ WSA draws on complexity science and complex adaptive systems which explain the ways in which factors and relationships interact and create sets of outcomes. The authors identified ten features of a WSA: 1) Identification of a system and its boundaries; 2) Capacity building; 3) Creativity and innovation; 4) Establishing relationships; 5) Engagement; 6) Establishing strong methods for communication across the system; 7) Embedding action and policies within organisations; 8) Developing leadership throughout the system; 9) Robustness and sustainability and 10) Monitoring and evaluation. Theoretical understandings of how WSAs work to produce anticipated outcomes is important to improve understanding of the processes involved in implementing a successful WSA for tackling obesity. Lack of theoretical underpinnings to the models used in implementing whole systems approaches could lead to difficulty when attempting to distinguish in what context a particular model would be most appropriate or effective and what processes it entails. This reduces the value of the accumulated evidence base as we are less able to identify characteristics or features that may contribute to the effectiveness of the implementation of WSAs in different settings. In order to address this, we undertook a review of reviews to identify evidence about the implementation of WSAs and the various approaches, theories or models used to underpin them. Given that several reviews have been conducted in this complex area of WSAs, a logical and appropriate next step was to conduct a review of reviews allowing the findings of separate reviews to be collated, compared, and contrasted, thereby providing public health policy makers evidence they need for the implementation of WSA to obesity, diet, and healthy weight.

### Aim

Our aim was to synthesize existing evidence reviews that investigate how whole systems approaches to diet and healthy weight have been implemented and evaluated nationally and internationally. This review of reviews sought to answer the following questions: 1. What models or theories (these are empirical frameworks, agent based, behaviour change models or statistical models) have been used to implement WSAs to diet, healthy weight, or obesity? 2. How have WSAs to diet, healthy weight or obesity been evaluated to date? 3. What evidence is there of the effectiveness of WSAs to diet, healthy weight, and obesity? and 4. What has been the contribution of the public and/or service users in the development of WSAs to diet, healthy weight, or obesity? Given that we were interested in a broad set of research questions about the current state of the WSA evidence base, rather than a single specific or precise question which would be typical of a standard systematic review, it was determined that a scoping review of reviews was the most appropriate methodological approach [18].

## Methods

A protocol for our review was published within a wider intervention study protocol [17]. The conduct of this review was based on the framework and principles reported by Arskey and O’Malley [19] and further developed by Levac et al. [20]. The review included the following five stages: 1) Identifying the research question; 2) Identifying relevant studies; 3) Study selection; 4) Charting the data; and 5) Collating, summarising, and reporting results. Arksey and O’Malley [19] reported an optional ‘consultation exercise’ which involves including practitioners and service users in the review to identify potential additional references for inclusion. Therefore, we included members of the public through our Public Involvement in Research group (PIRg), where four members actively engaged in all five stages after receiving training in conducting systematic reviews. See Smith et al. [21] for guidance on involving service users in conducting systematic reviews.

### Information sources and search strategy

The following five databases were searched from 1995 to 2022 to identify eligible studies for the review: CINAHL, Scopus, PsycINFO (ProQuest), the Cochrane Library, and MEDLINE. This timeframe was deemed sufficiently wide (12 years before the Foresight report and its accompanying complex systems map of obesity [22, 23]) to include all relevant evidence. The search strategy included a combination of keywords and database-specific terms, including the medical subject headings (MeSH) that cover WSAs in promoting diet and healthy weight: diet, nutrition, malnutrition, eating habit, eating behaviour, food choice, unhealthy diet, healthy weight, unhealthy weight, obesity, obese, overweight, underweight, fat, body fat, body mass index, body weight, physically inactive, physically active, physical inactivity; whole system approach and related terms such as systems approach, system modelling, collaborative working, joint working, multiagency, multiagency working, interagency, interprofessional collaboration, interprofessional teamwork, and community wide. These terms were modified to meet the search requirements of each database. Included papers were those reporting WSAs and their implementation, to identify the levers and opportunities influencing this. In addition, reference sections of identified articles were scrutinised for additional relevant articles.

### Eligibility criteria

Review papers were included if they satisfied all the following eligibility criteria: a review paper (narrative, scoping, systematic or meta-analytic); available in English language; published between 1995-March 2022; focus of the review was on the application of a WSA to diet, healthy weight, or obesity; reported the approach, theory, model or framework used to implement a WSA. Papers that did not meet these criteria were excluded from the review.

### Selection of reviews

**Fig 1:**
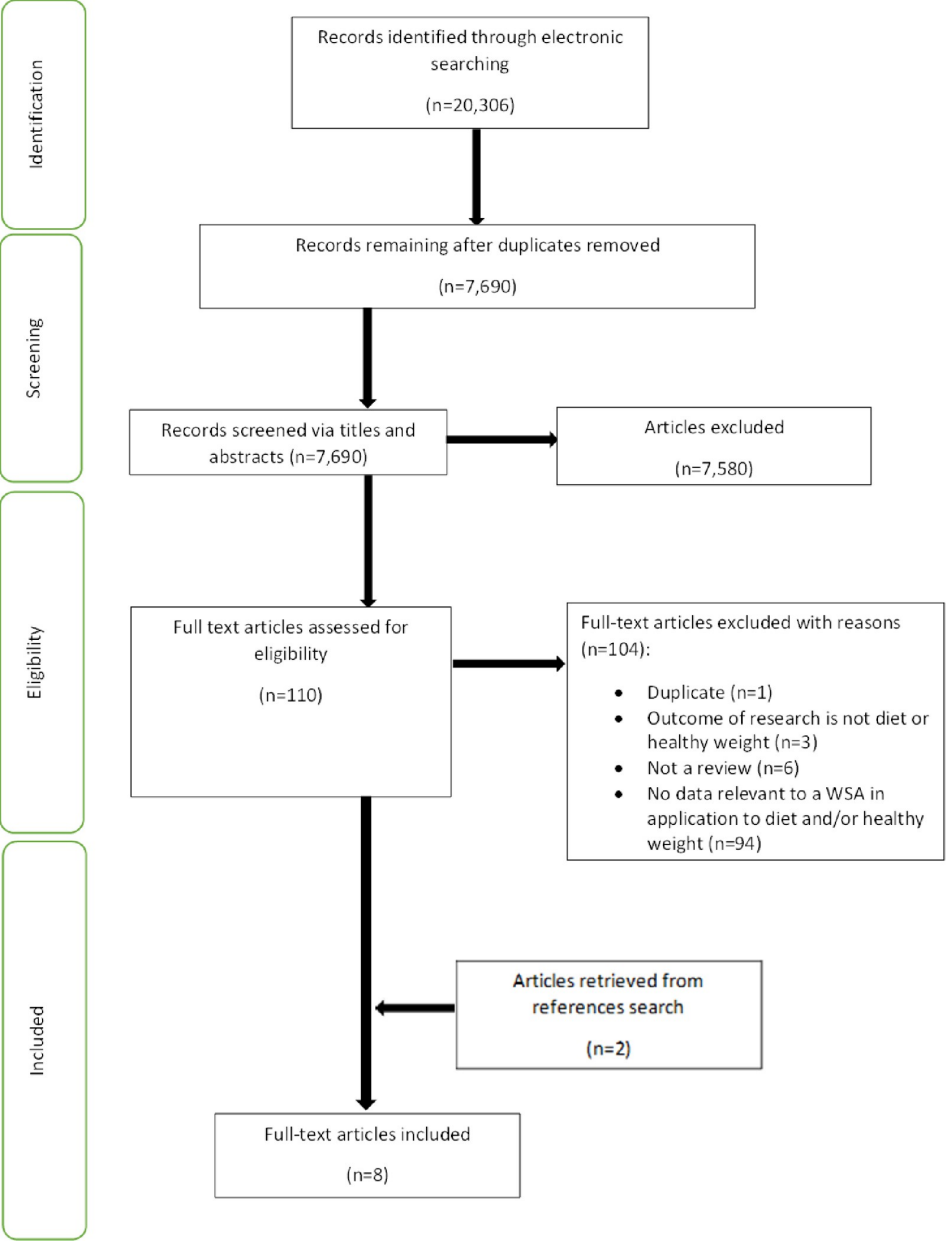
PRISMA flow diagram illustrating the search strategy.

The results from the literature searches were exported into Covidence, a web-based screening and data extraction tool [24]. Duplicate articles were removed. The review team comprised of 13 members of the wider PHIRST research team. Eleven (JI, CB, NL, OF, JJ, IF, KBa, GB, WW, KBr, AW) members of the research team screened the title and abstracts to determine each article’s eligibility for full-text screening based on the eligibility criteria. The PIRg, part of the NIHR-funded PHIRST Connect team, were included in the review team (JJ, SH, KBa, GB) and participated in the title and abstract screening, as well as full-text review to identify eligible articles [25]. Reviewers met throughout the screening process to resolve conflicts and discuss uncertainties related to study selection [20]. The Preferred Reporting Items for Systematic Reviews and Meta-Analysis (PRISMA) chart (see Fig 1) reports the phases of article identification and selection [26].

Full-text screening was carried out independently. A data extraction form was developed by the authors to confirm relevance and to extract study characteristics such as: author information (title, author name and year of publication), aims and objectives of the review, type of review (e.g. systematic, rapid etc.), PICO (population, intervention, comparator and outcomes as seen in systematic reviews), inclusion criteria (where appropriate), models or theories used to implement WSAs, evidence of the effectiveness of WSAs, and public/service user contribution in the development of the WSA. This form was reviewed by the research team and piloted by all reviewers before implementation, resulting in minor modifications to the standardised form. Reviewers OAF and CB met to resolve any conflicts, and to ensure consistency between reviewers and the research question/purpose [20]. OAF mapped the initial process of data extraction onto the form. GB, KB and HG reviewed and corroborated the extracted data on the form, ensuring that all relevant data was included. Articles were excluded at this phase if they were found on detailed inspection, to not meet the eligibility criteria (see Fig 1). Some challenges were experienced in applying the inclusion criteria as the term WSA was relatively new, and some studies although applying a WSA may have been referred to differently. This concern was also evident when examining what model of a WSA was implemented and subsequently evaluated. Both challenges relate to the various terms and definitions that are used for WSA, a consideration raised in previous reviews [16].

## Results

Electronic searches identified 20,306 articles, resulting in 7,690 unique articles to be screened for inclusion after duplicates were removed (see Fig 1). Titles and abstracts were assessed for their relevance to the review based on the inclusion criteria: 7,580 were excluded resulting in 110 articles being retained for full-text screening. Full texts of these articles were obtained and after applying the inclusion criteria, 105 articles were excluded; one article was a duplicate, three articles did not address diet and/or healthy weight and/or obesity, six articles were not reviews; 94 articles did not provide data relevant to a whole system approach to diet and/or healthy weight and/or obesity; two articles were retrieved from references list searches of the six remaining articles. This left eight review articles written in English, published between 1995 and March 2022 which focused on the application of a WSA to diet, healthy weight, or obesity and in all but one case, reported an approach, theory, or model of a WSA. Characteristics of the included reviews are shown in Table 1. Of the eight reviews identified, one was an umbrella review [27], four were systematic reviews [16, 28–30], one a rapid review [12] and two were literature reviews, one of which was on strategic reports written for government and public health policy [31] and the other focused on obesity, prevention and interventions in pregnant women, infants, children, and adolescents [32].

**Table 1:** Descriptive and PICO characteristics of included reviews.

No	Authors (date) and Title of the article	Aims/objectives	Review type	Population (where reported)	Intervention (where reported)	Study design and Comparator (where reported)	Outcomes (where reported)	Overall Conclusion
1	Sawyer, Lenthe, Kamphuis, Terragni, Roos, Poelman, Nicolaou, Waterlander, Djojosoeparto, Scheidmeir, Neumann-Podczaska and Stronks [27] Dynamics of the complex food environment underlying dietary intake in low-income groups: a systems map of associations extracted from a systematic umbrella literature review.	To develop and apply novel causal loop diagramming methods to construct an evidence-based map of the underlying system of environmental factors that drives dietary intake in low-income groups.	Systematic umbrella review	Low-income youths and adults in high/upper-middle income countries	Data was extracted from 43 review articles and after expert consensus to develop an evidenced based systems dynamics causal model of dietary intake	Information on the determinants and associations between economic, social, physical, and political food environment on dietary intake	The dietary intake system operates within an economic paradigm based on supply and demand. The system incorporates five sub-systems: 1. Geographical accessibility; 2. Household finances; 3. Household resources; 4. Individual influences; and 5. Social and cultural influences. Causal loop diagram comprising 60 variables, conveyed goals which undermine healthy dietary intake.	To target dietary intake, innovative strategies that incorporate causal loop diagrams and a systems paradigm are required to target the complexities of the accessibility, affordability, and acceptability of unhealthy foods.
2	Bagnall, Radley, Jones, Gately, Nobles, Dijk, Blackshaw, Montel, and Sahota [16] Whole systems approaches to obesity and other complex public health challenges: a systematic review	To undertake a systematic review of national and international published evidence on WSAs targeting obesity, other public health areas and areas outside public health (such as social care, crime, and justice) To understand what is known about WSAs and how they can be implemented in practice	Systematic review	Any population where a WSA has been used, at local, regional, national, and international level	WSAs, defined as those that: -Consider, in concert, the multifactorial drivers of overweight and obesity, as outlined by Foresight, public health or the social determinants of health; Involve transformative co-ordinated action (including policies, strategies, practices) across a broad range of disciplines and stakeholders, including partners outside traditional health sectors; Operate across all levels of governance, including the local level so that such approaches are reinforced and sustained, and Identify and target opportunities throughout the life course (from infancy to old age)	Primary research or evaluation studies including RCTs, non-RCTs, natural experiments, before and after intervention studies, mixed methods evaluations (including case study), process evaluations (qualitative and mixed methods); cost-effectiveness, cost-benefit, and cost-utility studies Comparator: Any or none	Health outcomes, e.g., weight, Body Mass Index (BMI), Type 2 Diabetes, diet and nutrition, physical activity, psychological well-being & quality of life; co-morbidities related to obesity, reductions in health inequalities, reductions in premature morbidity and mortality, cardiovascular disease, and obesity-related cancers. Organisational outcomes e.g., cross-sector collaboration; new partnerships; environmental changes; resource allocation; leadership etc. Process outcomes, e.g., what each project aimed to achieve and barriers and facilitating factors associated with achieving or not achieving those aims. Outcomes may be at individual, local, regional, or national/ federal/ principality level. Process and implementation outcomes e.g., training, recruitment, sustainability, people’s views on barriers and facilitators to implementation of WSAs. Cost, cost-effectiveness, cost-benefit, or cost-utility.	Systems approaches to tackle obesity can have benefit, but evidence of how to operationalise a WSA to address public health problems is still in its infancy. Not all ten features of a WSA identified by Garside et al. [15] are needed to achieve positive changes in health outcomes
3	Bleich et al. [28] A Systematic review of community-based childhood obesity prevention studies	To conduct a systematic review that included community-based childhood obesity prevention studies in high income countries where the interventions were delivered in a community where there was a change of policy.	Systematic review	Children	Childhood obesity prevention studies in high income countries	RCT, non RCTs, quasi- experimental.	A total of nine studies were included; five were RCTs and four non–RCTs. Five studies were conducted in the community setting in combination with at least one other setting such as the home, three were conducted in. the community and schools, and one study was conducted only in the community. BMI or BMI z-scores were reported in four of the nine studies. Two studies reported significant. Improvements in physical activity and one in vegetable intake.	Evidence is moderately convincing that diet and physical. Activity interventions conducted in the community with a school component are more effective at preventing obesity. A high level of variability across research designs may have led to inconsistent outcome findings.
4	Langellier, Bilal, Montes, Meisel, Cardoso, de Oliveira Cardoso, and Hammond [29] Complex Systems Approaches to Diet: A Systematic Review	To conduct a systematic review of studies that have used Agent Based Models (ABM) or Systems Dynamic Models (SDM) to understand the complex systems that influence population diet, with particular emphasis on identifying the complex system structures explored and methods used by each study.	Systematic review	Models were generated using available empirical population data. Twenty-two ABM studies and five SDM studies were included.	Data extracted from each study either included ABM or SDMs that included purpose, diet and nutrition outcomes, integration with empirical evidence, and model design elements.	Simulation models using empirical epidemiological data	Factors that influenced diet in the complex system were at neighbourhood- (e.g., residential segregation), interpersonal- (e.g., social influence) and individual-levels (e.g., food purchasing decisions). Food pricing, food environment, advertising, nutrition labels, and social norms influenced policy decisions.	Complex systems approaches to diet and nutrition can be used to understand mechanisms driving population-level diet, increasing use of models for policy decision support, and leveraging the wide availability of epidemiologic and policy evaluation data to improve model validation.
5	Skinner and Foster [30] Systems Science and Childhood Obesity: A Systematic Review and New Directions	To conduct a systematic literature review of studies that used systems science methodologies to study obesity in the context of public health.	Systematic review	Types of models used in systems science	Systems science models to obesity	Studies must examine obesity in the framework of systems Science, include original analyses, rather than discussing only how systems science could be used, and must include obesity in the model, as a predictor and/or outcome.	The 21 included studies addressed four areas of systems science in obesity: (1) translating interventions to a large scale, (2) the effect of obesity on other health or economic outcomes, (3) effect of geography on obesity, and (4) the effect of social networks on obesity.	Further research is required on applying systems science to childhood obesity that will require multidiscipline involvement, and skills from social scientists, clinical scientists, public health researchers, and researchers with systems science knowledge.
6	Safefood [12] Whole Systems Approach to childhood obesity: A review of the evidence	A rapid review of WSAs to obesity to support policy makers and local decision makers to progress understanding of WSAs to childhood obesity.	Rapid Review	Children	Studies that included a WSA to childhood obesity.	Study of any type.	14 studies were identified. Three studies reported positive outcomes in healthy behaviours and/ or anthropometric measures such as improvements in BMI and BMI z-score. Seven studies reported mixed findings; three negative outcomes; and one study was on-going at the time of the review, but has subsequently been reported [40]	When implementing a WSA to obesity prevention, political structures, visibility of leadership, cross-departmental government policy alignment; flexible local approaches that target health inequalities; increased community involvement and engagement; and long-term funding and evaluation are required.
7	Johnston, Matteson and Finegood [31] Systems science and obesity policy- a novel framework for analysing and rethinking population-level planning.	To demonstrate the use of a systems-based framework to assess solutions to complex health problems such as obesity. To explore the utility of the Intervention level framework (ILF) by identifying a rich set of recommendations garnered for a variety of purposes from decision-makers working in different environmental contexts. Identify using ILF how best to act in addressing the complex problem of obesity. What are the various system levels and the specific interventions required to support large-scale change.	Literature Review of strategies, policies, and academic evidence.	9 strategies or reports written by or for governments or health authorities in the United States and Canada, 1 Cochrane review of interventions to prevent childhood obesity, and 2 reports produced by the Institute of Medicine (IOM). 7 reports focused on childhood obesity.	Used the ILF framework to code strategies for tackling obesity within each of the 12 selected policy docs/systematic review according to which level of the system they were operating at; 1) Paradigm (deepest held beliefs) a sort of ethos of an approach e.g. adopt a holistic view of health; 2) Goals (or targets) e.g. achieve a childhood obesity rate of 5%; 3) Structure (across the system) e.g. recommendations about cross-sectoral collaborations such as food producers and schools; 4) Feedback loops and delays (loop dynamics) which involves collecting and analysing data about the relationships between different factors in the system and how changes in one may influence or cause changes elsewhere e.g. evaluate the effect of a new tax on unhealthy foods; or 5) Structural elements (subsystem specific) e.g. improving food and physical activity environments and running health promotion campaigns targeted at particular groups.	N/A	N/A	ILF provides a template to encourage systems thinking and more strategic policy design that goes beyond the focus on individual responsibility alone to target obesity.
8	Nader, Huang, Gahagan, Kumanyika, Hammond and Christoffel [32] Next steps in obesity prevention–altering early life systems to support healthy parents, infants, and toddlers	To combine multidisciplinary expert understanding of the strengths and weaknesses of current approaches to the prevention of childhood obesity. To support the need for and refine a broader early life cycle approach to the prevention of childhood obesity.	Expert panel discussion and targeted literature review	Pregnant women, infants, children, and adolescents	N/A	N/A	N/A	A twin approach to tackling obesity is proposed: (1) Intervention is necessary before, during, and after pregnancy, and for very young children, and (2) systems approaches are needed for sustainable, prevention of childhood obesity and its consequences.

The criteria relating to reporting the approach, theory or model used to implement a WSA was found to be challenging to apply. Our searches identified three different types of review which we have set out below showing three different ways in which theories, models, or frameworks for a WSA have been applied in or emerged from the review process. A narrative synthesis approach is applied to address each of the four research questions.

### Review type 1: Reviews that include studies that have evaluated the implementation of WSAs to obesity

The Bagnall et al. [16] review is a systematic review of studies that have evaluated WSAs to obesity and other complex health challenges. It used the ten features of a WSA set out by Garside et al. [15] to identify the extent to which interventions featured in articles that met their inclusion criteria could be said to be taking a WSA approach to healthy weight/obesity. The review included 65 studies of which 33 focused on obesity. Findings showed improved health outcomes such as reductions in Body Mass Index (BMI), increased parental and community awareness, community capacity building, nutrition and physical activity environment changes, and improved safety and wellbeing of community members [16]. Of the 33 obesity focused studies, 13 included all ten features outlined within Garside et al [15]. Of those 13 studies, five reported health or wellbeing outcomes, for example, consumption of sugary drinks, changes to BMI, changes to physical activity. Two reported outcomes that were associated with the social determinants of health (social economic factors), while eleven reported mainly process outcomes. The review suggests that all ten features of a WSA were not required to achieve changes in health. Improvements in nutrition and physical activity were shown in an evaluation of the Central California Regional Obesity Prevention Program [33], which met all ten WSA guidance features. BMI, parental awareness, and community capacity building was improved in an evaluation of the Romp and Chomp programme in Australia [34, 35], which met nine out of the ten WSA guidance features. BMI was also improved in two non-RCTs of Be Active Eat Well (BAEW) in Australia [36, 37] which met seven out of the ten WSA features and ‘Shape up Somerville’ an intervention in the USA [38], which met eight out of the ten guidance features. Improvements in fitness and BMI z-scores (BMI z score is a measure of how many standard deviations a child or young person’s BMI is above or below the average BMI for their age and gender) were observed in a prospective cohort study of Healthy Living Cambridge Kids in the USA, which met four of the ten WSA features [39]. Taken collectively it would appear that having all ten features of the WSA guidance is beneficial but not always necessary to lead to improvement in BMI or reduction in obesity. The success of a WSA was attributed to some key facilitators. These included the full engagement of stakeholders, good governance, trust and capacity, sufficient time to build relationships, sufficient finance, and the embedding of the WSA within broader policy so that other less obvious policy changes may also impact obesity (e.g., town planning). This meant ensuring that WSA principles were established within the objectives of individual organisations, rather than being something organisations were required to do in addition to their core work. Although initial findings are promising, a cautionary approach was advised when advocating the benefits of a WSA. Many study descriptions of what constitute a WSA, and the outcomes reported in some studies, were limited, or lacked longitudinal follow-up. Furthermore, consistency in definition, application, and thorough evaluation of WSAs was lacking.

Similar to Bagnall et al, the rapid review of WSAs to obesity prevention commissioned by Safefood published in 2021 [12] included evaluations of WSAs tackling obesity and aimed to progress understanding of WSAs to childhood obesity. It included reference to the Garside et al [15] ten features of a WSA and the 10 pillars of the Amsterdam Healthy Weight programme [40] in its assessment of the evidence. The review identified 14 studies that explored WSAs to obesity. Most interventions involved a town or city-level community intervention, with varying durations. Three of the 14 studies reported positive outcomes in health behaviours and/ or anthropometric measures such as improvements in BMI and BMI z-score. These studies included the ‘Healthy Living Cambridge Kids USA’ [39], ‘Romp and Chomp’ [34, 35] and ‘Shape Up Somerville’ [38] initiatives (similar to the Bagnall et al. study above [16]), all were deemed to have strong to moderate methodological quality. Seven studies reported mixed findings on the effectiveness of WSA; three reported negative outcomes; and, one study was on-going at the time of the review, the Amsterdam Healthy Weight Approach (AHWA) in the Netherlands but has subsequently been reported [41]. For the AHWA a logic framework was developed describing the mechanisms underpinning a WSA to childhood overweight and obesity prevention [41]. The aim of the logic framework was to inform further AHWA development, monitoring, and evaluation and to promote lessons learned and share understanding of the wider working principles of WSAs in public health to others embarking on WSAs. The Safefood [12] review identified that facilitators to implementing a WSA were: the need for strong leadership; allocating sufficient time to building relationships; community involvement and capacity building; ensuring consistency in language of WSA across sectors and allocating adequate time, resource, and financial support. Funding and evaluation commitments using existing population and health surveillance data to implement a WSA to preventing childhood obesity was also recommended.

Bleich et al’s [28] systematic review included nine studies, given the year of publication and when WSA was not as commonly used it was no surprise that WSA was not mentioned. The authors did not make reference to a theory, model, or framework of a WSA in their assessment of the literature, but it was retained within our scoping review of reviews because it is one of only three reviews identified where evaluations of the implementation of a WSA to obesity have been included, all studies were community based and some were in response to a policy change [12, 16, 28]. All studies were from high income countries. Some encouraging findings were shown for improvement in BMI/BMI z-scores. A total of nine studies were included; five were randomized controlled trials (RCTs) and four were non–RCTs. Five studies were conducted in the community setting in combination with at least one other setting such as the home, three were conducted in the community and schools, and one study was conducted only in the community. BMI or BMI z-scores were reported in four of the nine studies. Two studies reported significant improvements in physical activity levels and one in vegetable intake. The authors concluded that evidence is moderately convincing that diet and physical activity interventions conducted in the community with a school component are more effective at preventing obesity. They also highlighted that the high level of variability across the research designs may have contributed to inconsistent findings. Notably, studies varied in their use of research design methodologies (e.g., RCTs vs quasi-experimental studies), were located in non-uniform settings, and used different intervention types to improve health (e.g., physical activity or combined diet and physical activity).

### Review type 2: Reviews that draw on evidence gathered to present a model, theory, or framework on which to base a WSA approach to diet, healthy weight, and obesity in the future

Nader et al. [32] conducted a literature review of 1,438 articles that focused on obesity, prevention and interventions in pregnant women, infants, children, and adolescents. The rationale for the focus on pregnancy and children was based on intervening as early as possible. They included review articles initially, then used these to refine their search for examining the early life cycle approach to the prevention of childhood obesity. It was not clear from what was reported how many articles met the final criteria for inclusion in the review. They reported that in a Cochrane review [42] article that obesity prevention interventions may produce the largest magnitude of effect early in life, while two Institute of Medicine (IOM) reports emphasized both the need for interventions early in life [43] and the use of a ‘systems perspective’ to fill in gaps in obesity research evidence that can more effectively guide policy [44]. They conclude from their review that early intervention and a systems approach need to be combined as the next logical step in tackling obesity. The authors propose a framework comprised of eight pathways to guide the implementation of a systems approach targeting obesity in early life. They identify that local, state, and national policies influence the physical environment (1) and the social environment (2). Policy also influences the health care system (3) and the physical and social environment influence each other (4). The physical and social environments influence family practices and individual behaviour (5) as does the healthcare system (6). The healthcare system also influences the physical and social environments (7) and finally family practices and individual behaviour also feedback and influence local, state, and national policies. They go on to identify a set of goals or targets for health across pregnancy, infancy and toddlerhood and provide examples of interventions that would be considered appropriate for each of the eight system pathways identified. They also go on to provide recommendations for how such a WSA should be evaluated in the future and conclude that taking their outlined approach and evaluating it are the next logical steps in tackling the obesity epidemic.

Skinner and Foster [30] conducted a systematic review to examine the causes and consequences of obesity using systems science. Systems science incorporates Systems Dynamic Modelling, Agent Based Modelling or discrete event simulation as an approach to look at complex social interventions. A total of 21 studies were included that addressed four areas in systems science. These included: 1) translating obesity interventions to a large scale; 2) Determining the association of obesity with other health or economic outcomes; 3) Reporting the effect of geography on obesity and; 4) The effect of social networks on obesity. The authors concluded that more specific complex models are required that map obesity from childhood into adulthood. In order to incorporate a systems model to obesity in children to adulthood there is a need to ensure there is multidisciplinary involvement, and skills from social scientists, clinical scientists, public health researchers, and researchers with systems science knowledge to input into the development of mapping the system.

Johnston et al. [31] conducted a review of systems science and obesity policy to provide a novel framework for analysing and rethinking population-level planning. Their aim was to demonstrate the use of a systems-based framework to assess solutions to complex health problems such as obesity. They explored the utility of the Intervention Level Framework (ILF) and identified a set of recommendations and specific interventions for decision-makers to implement ILFs to obesity for large scale behaviour change [45]. To achieve this, they reviewed twelve reports published between 2004 and 2013, these consisted of nine strategies or reports written by or for governments or health authorities in the United States and Canada, one Cochrane review of interventions to prevent childhood obesity, and two reports produced by the Institute of Medicine (IOM). They used the ILF framework to code the recommended strategies (or interventions) for tackling obesity within each of the 12 selected reports according to which level of the system they were operating: 1) Paradigm (deepest held beliefs or the ethos of an approach e.g. adopt a holistic view of health); 2) Goals (or targets) e.g. achieve a childhood obesity rate of 5%; 3) Structure (across the system) e.g. recommendations about cross-sectoral collaborations such as food producers and schools; 4) Feedback loops and delays (loop dynamics) which involves collecting and analysing data about the relationships between different factors in the system and how changes in one may influence or cause changes elsewhere e.g. evaluate the effect of a new tax on unhealthy foods; or 5) Structural elements (subsystem specific) e.g. improving food and physical activity environments and running health promotion campaigns targeted at particular groups. They also coded recommended strategies based on variables listed in the Foresight Obesity systems map [23] forming a taxonomy of 30 variables organized around four subsystems; 1) social and individual psychology; 2) food production and consumption; 3) physiology and clinical care; and 4) physical activity. They found that the majority of strategies focused on altering the determinants of energy imbalance through targeting food intake and physical activity at an individual level and that 76% of recommended strategies were Structural (i.e., ILF level 5). The findings suggest there is considerable scope for increasing the systems focus of policy design to address obesity. They concluded that the ILF provides a template to encourage systems thinking and more strategic policy design grounded in complexity science that goes beyond the focus on individual responsibility alone.

### Review type 3: Reviews that refer to systems dynamic models, causal loop diagrams and agent based models

Langellier et al. [29] conducted a systematic review of complex systems approaches to diet using Agent Based Models (ABMs) and System Dynamic Models (SDM) incorporating epidemiological data. A total of twenty-seven studies were included; 22 ABM studies and five SDM studies. Factors that influenced diet in the complex system were neighbourhood (e.g., residential segregation), interpersonal (e.g., social influence) and individual level (e.g., food purchasing decisions), while food pricing, food environment, advertising, nutrition labels, and social norms influenced policy decisions. For those using SDM, several studies used longitudinal data to estimate the values of parameters related to either the population (i.e., initialized the population based on demographic and health data from the baseline observation of a cohort study) or processes under investigation. Most studies used empirical data to inform values of key parameters for each of the models, however there was variation in approaches to model calibration and validation. The authors recommended that integrating complex systems modelling like SDM with policy intervention research shows promise for preventing obesity. They suggested that the Childhood Obesity Modelling for Prevention and Community (COMPACT) study is an example of where systems approaches are linked with community obesity interventions where the model can be updated as new implementation and evaluation data become available, it is then that the intervention can be refined to get the optimal outcome [46, 47].

Sawyer et al. [27] conducted a recent umbrella systematic review of the dynamics of the complex food environment underlying dietary intake in low-income groups and produced a data informed systems map based on the 43 reviews included. Studies were extracted if they reported the economic, social, physical, and political food environment associations with dietary intake. The authors reported that the dietary intake system operated within an economic paradigm based on supply and demand, and that the system incorporates five sub-systems: 1) geographical accessibility; 2) household finances; 3) household resources; 4) individual influences; and 5) social and cultural influences. A causal loop diagram comprising 60 variables was reported. Their findings revealed how poor dietary intake in low-income groups can sustain a food environment that increases the accessibility, availability, affordability, and acceptability of unhealthy foods. They proposed that in order to reshape system dynamics that are currently driving unhealthy food environments, innovative strategies are needed to facilitate longer-term management of household finances and socially-oriented practices around healthy food production, supply, and intake. The diagram in the Sawyer et al. [27] article (they cite as Fig 2) conveyed a map of the system illustrating that disruption to the key feedback loop of ‘demand’ for unhealthy food is necessary, and that to influence this, simultaneous interventions targeting the supply of food, household finances and resources, and social, cultural, and individual influences (part of people’s ‘lived experiences’) are likely needed.

### RQ1. What models, theories, or frameworks have been used to implement WSAs to obesity?

The models, theories, or frameworks that have been used within reviews are summarised in Table 2 below. These include systems science that incorporate mathematical simulations, such as Agent Based Models and Systems Dynamics Models for mapping the complex system with key features of the system and feedback loops to show how system components links together [30]. According to Skinner and Foster [30] there is a need for multidisciplinary involvement in mapping a system and more specific models that represent diet and healthy weight in children. In Langellier et al.’s [29] systematic review of complex systems approaches, twenty-seven studies highlighted that neighbourhood- (e.g., residential segregation), interpersonal- (e.g., social influence) and individual-level (e.g., food purchasing decisions) factors influenced diet, while food pricing, food environment, advertising, nutrition labels, and social norms influenced policy decisions. Encouragingly there were some longitudinal studies reported in their review. The most recent review was conducted by Sawyer et al. [27] and involved 43 studies that described economic supply and demand factors as the main components underpinning the obesity system. A total of 60 variables were reported and a systems map that could be applied to areas of low income. Four A‘s was described as influencing unhealthy food choice: accessibility; availability; affordability; and acceptability of unhealthy foods. Encouraged in Sawyer et al.’s [27] review was the incorporation of lived experience of the participants, a key feature of public health research in recent years, and that will likely only be more prominent in the design and evaluation of public health research into obesity going forwards [25]. In Bagnall et al.’s [16] review they described articles based on the extent to which they included Garside et al.’s [15] key features of a WSA, although not a theory, this list of features can provide a useful checklist for those developing and implementing WSAs. Thirteen of the 33 studies that were focussed on WSAs to obesity included some of the key features. Findings suggest that all ten features were not required for implementation of a WSA to show effectiveness. Key WSA facilitators according to Bagnall and colleagues were full engagement of stakeholders, good governance, trust and capacity, sufficient time to build relationships, sufficient finance, and the embedding of the WSA within broader policy so that other less obvious policy changes may also impact obesity. In the review conducted by Safefood [12] they described the Amsterdam Healthy Weight Approach (AHWA), and a AHWA logic framework [41] that has shown merit in WSA development, monitoring and evaluation.

**Table 2:** Identifying the various models and features extracted from the review articles to describe or support a systems approach to diet, healthy weight, and obesity.

Various models and features described as a WSA
Amsterdam Healthy Weight Approach (AHWA) [12]
Amsterdam Healthy Weight Approach Logic Framework (AHWA-LF) [12]
Agent Based Model (ABM) [30]
ANGELO (Analysis Grid for Environments Linked to Obesity) Framework [12]
Childhood Obesity Modelling for Prevention and Community Transformation (COMPACT) [29]
Discrete Event Simulation (DES) [30]
Intervention-Level Framework (ILF) [31]
NICE and Garside’s list of 10 key features of a WSA [16]
System Dynamics Model (SDM) [30]
Systems Framework to Prevent Obesity by Targeting Early Life [32]
Systems Science Properties and Nine Properties of Obesity [30]

Because the models, theories and frameworks identified and extracted at the review level and included in Table 2 above were either an outcome of the review process or typically used to assess the included literature we also considered the primary studies within the three reviews that focused on the implementation and evaluation studies of WSAs [12, 16, 28]. In Table 3 below we present data extracted from each of the primary studies on which review it came from and the quality rating provided for the study by that review (as applicable; some studies are in more than one review; some in only one); the model, theory or framework (if any) used to develop and implement the WSA (RQ1), the study designs used to evaluate the WSA (RQ2), what the reviews said about the effectiveness of the WSA (RQ3) and any information identified about whether and how the public or end-users were involved in developing, implementing or evaluating the WSA (RQ4).

**Table 3:** Table presenting primary studies included across both Bagnall et al. [16], Safefood [12], and Bleich et al. [28] reviews arranged by review ranked study quality (highest quality first).

Study	Included in Bagnall et al? If yes, quality rating?	Included in Safefood? If yes, quality rating?	Included in Bleich et al? Doesn’t include quality rating by article	What models theories or frameworks have been used to implement WSAs (RQ1)	Study design (RQ2)	Evidence of effectiveness? (RQ3)	Public involvement (RQ4)?
Chomitz et al. 2010 (Cambridge, USA) ‘Healthy Living Cambridge’ [39]	Yes; moderate	Yes; 8.1 strong	Yes	Drew on the socioecological model (McLeroy et al. 1988) to target community, school, family, and individuals. Met 4/10 WSA NICE guidance features (Bagnall et al)	Prospective cohort study	Increase in physical fitness +14.6% and decrease in BMI and BMI z scores (Safefood). Positive effects on fitness and obesity (Bagnall et al) Positive significant effect on change in BMI from baseline and BMI z-score (Bleich et al)	Used a Community-based Participatory Research (CBPR) Approach to build the intervention and run the evaluation over ten years
Sanigorski et al. 2008 (Colac, Australia) ‘Be Active Eat Well’. [37]	Yes; good	Yes; 7.9 moderate	No	Their approach is defined as a capacity-building approach (Hawe et al. (1997) Met 7/10 NICE guidance features	Non-RCT	Safefood report increased BMI z scores (i.e., negative effect) Bagnall et al. in contrast report positive effects on BMI and health behaviour Children in the intervention gained less weight, had lower increases in waist circumference, lower increases in BMI z-score and waist to height ratio; but no sig difference between groups in incidence of overweight/obesity	BAEW was designed to build the community’s capacity to create its own solutions to promoting healthy eating, physical activity, and Healthy weight in children aged 4–12 years.
Economos et al. 2007 (Somerville, USA) ‘Shape Up Somerville’ [38]	Yes; moderate to good	Yes; 7.9 moderate	Yes	No framework, theory, or model of a WSA mentioned but the WSA was developed using community-based participatory research. The focus was on changing a range of food and physical activity environments for children across the day. Met 8/10 NICE guidance features	Non-RCT	Decrease in BMI z scores (Safefood) Positive effect on BMI (Bagnall et al) Desirable intervention effect on BMI change from baseline and BMI z score (Bleich et al)	Using a community-based participatory research (CBPR) collaborative approach researchers partnered with community members of three culturally diverse urban communities to conduct a controlled trial evaluating environmental change effect on BMI z-scores of young children. Engaged with wide variety of community members to plan the study.
Johnson et al. 2012 (Colac, Australia) ‘Be Active Eat Well’. [36]	Yes; good	No	No	As above (Sanigorski et al.) Their approach is defined as a capacity-building approach (Hawe et al. (1997) Met 7/10 NICE guidance features	Non-RCT	Bagnall et al. reported positive effects on BMI and health behaviour	Used data from BAEW (Sanigorski, 2008) which was designed to build the community’s capacity to create its own solutions.
De Henauw et al. 2015 (8 European Union countries) ‘IDEFICS’ [48]	No	Yes; 7.9 moderate (top end)	No	Developed with an Ecological Health approach	Mixed methods involving observational cohort study and non-R clusterCT	Increases in BMI z scores, fatness, body fat mass and waist/hip in children	No evidence in findings papers or other intervention materials of public or end-user involvement of the development or delivery of the IDEFICS intervention; though it was culturally adapted in each country
Verbestel et al. 2015 (8 EU countries) ‘IDEFICS’ [51]	No	Yes; 7.9 moderate (top end)	No	The Intervention Mapping protocol was used (Bartholomew et al. 2006)	Non-randomized cluster-controlled trial	Decrease in PA and increase in sedentary time	No mention of end-users or the public being consulted as part of intervention mapping
De Silva-Sanigorski et al. 2010 (Geelong, Australia) ‘Romp and Chomp’ [35]	Yes; moderate to poor	Yes; 7.5 moderate	Yes	No theory, model, or framework for the WSA approach mentioned. Met 9/10 NICE WSA guidance features	Mixed methods	Increases in vegetable servings per day and increases in activity; decreases in BMI in 3.5-year-olds. (Safefood) Positive effects on BMI, parental awareness, and community capacity building (Bagnall et al) Desirable intervention effect on BMI change (Bleich et al)	This study used extensive community consultation and stakeholder engagement and a management committee of stakeholders oversaw its implementation.
De Groot et al. 2010 (Geelong, Australia) ‘Romp & Chomp’ [34]	Yes; moderate to poor	No	No	No theory, model, or framework for the WSA approach mentioned. Met 9/10 NICE WSA guidance features	Mixed methods	Positive effects on BMI, parental awareness, and community capacity building (Bagnall)	Used the data from the ‘Romp & chomp’ study to evaluate the community capacity change. The ‘romp & chomp’ study used extensive community consultation, stakeholder engagement and was overseen by a management committee of stakeholders.
Bell et al. 2019 (South Australia) ‘OPAL’ [49]	No	Yes; 7.5 moderate	No	Informed by social marketing, community development and social ecological systems theory. Reference is made to National Health and Medical Research Council. A modelling system to inform the revision of the Australian Guide to Healthy Eating. Canberra: Commonwealth of Australia; 2011.	Quasi-experimental repeated cross-sectional design	No significant changes over time on multiple anthropometric measurements or BMI z score, multiple health behaviours including fruit and vegetable intake, nor health related quality of life	This study used centrally coordinated materials produced with community stakeholders and as each programme was shaped by the community’s needs they were different for each community.
Copeland et al. 2011 (Sheffield, UK) Change4Life/Healthy Towns (Sheffield, UK) Change4Life/Healthy Towns [55]	Yes; moderate to poor	Yes; 6.3 moderate	No	Drew on Foresight obesity map EPODE, ‘Be Active Eat Well’ and ‘We Can!’ interventions to identify a range of interventions. Met all 10 features of WSA NICE guidance	Nested case control study	Mixed effects on health and wellbeing outcomes with no reduction in obesity prevalence (Bagnall et al)	Some indication that there was community involvement due to the ‘what works’ report of ‐ Bringing professionals and practitioners together through a shared vision. Partnerships formed between individuals, organisations and communities which would not have happened if not for the systems approach.
Mead et al. 2013 (Nunavet & Northwest Territories, Canada)	No	Yes; 7.5 moderate	No	Developed in part from theory ‐ including, social cognitive theory and social ecological models	Pre-post questionnaires	Increases in health knowledge and behaviour and healthy intentions; decrease in unhealthy food consumption; no significant change to BMI (but unclear)	Yes–public involvement in intervention plan and materials. Interviews and public workshops and participatory methods employed in intervention development.
Raine et al. 2013 (Alberta, Canada) [57]	No	Yes; 7.9 moderate (top end)	No	ANGELO–Analysis Grid for Environments Linked to Obesity framework (Swinburn et al. 1999)	Pre-post surveys from intervention communities compared to comparator national survey data	No significant changes in chronic disease risk, BMI, or blood pressure	Yes– 4 unique communities defined and worked to address priorities relevant to environmental and social determinants of obesity
Vinck et al. 2016 (Mouscron and Marche-en-Famenne, Belgium) [58]	No	Yes; 7.9 moderate (top end)	No	None mentioned	Results weren’t compared against control. Quasi experimental design	No significant change in BMI	VIASANO project is based on the EPODE (‘Ensemble Prévenons l’Obésité Des Enfants’) methodology which is a community-based programme that included broad environmental strategies to stimulate long-term, sustainable behavioural change and prevent childhood obesity called.
Hoelscher et al. 2010 (Texas, USA) Travis County CATCH [60]	Yes; moderate	No	No	No specific programme or WSA is being looked at; the evaluation focuses on any and all policies and programmes that have been implemented in Texas and is focussed on collecting better data to evaluate the impact it is all having. No features of the WSA NICE guidance	Non-randomised trial	Mixed effects for obesity prevalence	Not mentioned
Department of Health 2010 (UK) Change4Life Redacted version [54]	Yes; moderate	No	No	Social Marketing part of the Healthy-Weight, Healthy Lives cross-governmental strategy for England Met 8/10 WSA guidance features	Mixed methods	Positive effects on parental health behaviour and awareness	This campaign involved everyone who had an interest in preventing obesity: teachers, healthcare professionals, community groups, businesses, charities, or individual members of the public. Also involved over 25,00 ‘local supporters’
Atalla et al. 2019 (Jaguariuna, Brazil) [59]	No	Yes; 6.3 low (top end)	No	None discussed	plausibility assessment	Increases in physical activity and leisure time physical activity; increases in recommended fruit and vegetable intake; decrease in BMI among those with overweight or obesity	None mentioned, interventions were based on government guidelines (SI2)
Schwarte et al. 2010 (California, USA) Central California Regional Obesity Prevention Program (CCROP) [33]	Yes; unclear quality	No	No	The CCROPP model of change Met 10/10 WSA NICE guidance features.	Unclear design	Positive effects on nutrition and physical activity environments	Community resident engagement in focus groups, resident surveyed, and elected stakeholders interviewed to determine priorities for action.
Gadsby et al. 2020 (Golborne, UK) [56]	No	Yes; 3.8 weak	No	The methodology and design was informed by the best practice principles for community-based obesity prevention developed in Australia, the World Health Organisation Good Practice Appraisal Tool, and the EPODE approach to childhood obesity prevention. The PESTEL framework (distinguishing political, economic, sociocultural, technological, and physical and legal environments) was used to explore and describe the influences that hinder or support the adoption of healthy lifestyles in the community. Theory of change was also used.	Non-experimental case study design and mixed methods	BMI only reported as not statistically significant–no detail; report increases in diet and increases in sedentary behaviour but reporting unclear	A programme plan was developed through stakeholder engagement
Amed et al. 2016 LIVE 5-2-1-0 Canada [50]	Yes; poor	No	No	Adapted version of the Re-AIM model called REFRAME model was designed. Also used Graham *et al*.’s knowledge to action (KTA) framework. The initiative is informed by the socio-ecological framework.	Mixed methods	Early indications of improvements in community awareness and action to promote healthy childhood behaviours (from stakeholder interviews)	Live 5-2-1-0 is a multi-sector initiative that works with a wide range of community stakeholders to share (through social marketing) and support (through systems level change; programmatic, environmental, and policy level) the evidence based Live 5-2-1-0 message. SCOPE, the organization that coordinates the Live 5-2-1-0 initiative. SCOPE’s knowledge exchange (KE) model emerged in its first phase of development where SCOPE established a partnership with a local backbone organization (*i*.*e*., local government, Division of Family Practice) in two pilot communities and through this partnership, a network of community-based, multi-sectoral partnerships was established via an intensive community engagement strategy that was rooted in the principles of community based participatory research
European Commission 2018 (Amsterdam, Netherlands) The Amsterdam Healthy Weight Programme	No	Yes; no data	No	Adaptation of the Dalghren & Whitehead Rainbow model and identification of 10 pillars of action mapped against the model	Workshop	Unpublished at time of Safefood review	None mentioned
Hawkes et al. 2017 (Amsterdam, Netherlands) The Amsterdam Healthy Weight Programme [40]	No	Yes; no data	No	Some early inspiration was drawn from the French EPODE [41] programme. Whole systems approach. 10 pillars of action of mapped against an adapted Dalhgren & Whitehead Rainbow model	Rapid response briefing paper	Unpublished at time of Safefood review but some mixed early findings	Collaborative working between paediatricians, GPs, healthcare professionals, parent and child professionals, youth health care nurses, youth counsellors, welfare professionals, and community organisations. Regular community engagement explore how programmes are working in practice and enables the council to respond to community needs and perceptions
Chang et al 2020 (Delaware, USA)	No	No	Yes	Used a ‘social-ecological’ strategy to promote 5-2-1-almost none	Quasi-experimental/non-randomised controlled trial	No significant change in the prevalence of obesity–argued it halted increases in obesity	None is mentioned–only use of strategic partnerships with child-focused agencies and organisations
Eiholzer et al 2010 [79]	No	No	Yes	Not a WSA. The study tested whether high-intensity training increases spontaneous physical activity in members of junior ice hockey teams	NA	NA	NA
Sallis et al 2003) (Sandiago, California) M-SPAN [61]	No	No	Yes	Cohen et al’s structural ecologic model of health behaviour targeting 1) availability of protective or harmful products & services 2) physical structures or characteristics of products or services 3) social structures and policies and 4) media & cultural messages	Cluster RCT	Desirable intervention effect of change in BMI in boys only	None is indicated
Singh et al 2009 (Netherlands) DOiT intervention [53]	No	No	Yes	Applied the Intervention mapping approach to WSA intervention development	Cluster RCT	No significant differences between groups on change in BMI	Another paper on the intervention mapping approach taken (Singh et al; 2006) does not indicate public involvement
Klesges et al 2010 (Memphis, Tennessee) Memphis GEMS [62]	No	No	Yes	Not a WSA–group behavioural counselling to promote healthy eating and increased physical activity	NA	NA	NA
Robinson et al 2010 (Oakland, California) Stanford GEMS [63]	No	No	Yes	Not a WSA–family-based dance classes and screen use reduction intervention	NA	NA	NA

Notably, those developing, implementing, and evaluating WSAs to obesity, healthy weight and/or diet have drawn on very different frameworks to guide their activities than those typically referenced in reviews of WSA evidence. Several papers reference use of socioecological theory to support them in considering appropriate interventions at the individual, family, community/school, and population/policy level [39, 48–50]. Several do not cite a model as such but describe having used some form of community capacity building or participatory research approach [36–38, 49]. Verbestel et al. [51] reports on the same WSA as De Henauw et al. [48] but reports use of the Intervention Mapping approach for the WSA development [52]. Singh et al [53] also report applying Intervention Mapping to the development of their WSA. Two studies refer to having taken a social marketing approach [49, 54]. Bell et al. [49] also make reference to a modelling system set out in a document authored by the National Health and Medical Research Council in Australia. EPODE was identified by three studies as having been drawn on for WSA development and implementation [40, 55, 56]. Additional models, theories and frameworks mentioned include the Foresight obesity map [55]; the ANGELO framework [57]; The CCROPP model [33]; an adaptation of the RE-AIM framework and the KTA framework [50]; and the 10 pillars of a WSA ]. Finally, Gadsby et al. [56] cite use of the best practice principles for community-based obesity prevention developed in Australia, the World Health Organisation Good Practice Appraisal Tool, and the PESTEL framework (distinguishing political, economic, sociocultural, technological, and physical and legal environments) in addition to EPODE. Four studies did not report any use of a model, theory or framework in the development or implementation of their WSA [34, 35, 58, 59]. Three studies included only within the Bleich et al review [28] were identified as not qualifying as a WSA (see Table 3) so no models are reported in relation to these.

### RQ2. How have WSAs to obesity been evaluated to date?

The study designs from the three reviews which focus on evaluation of the implementation of a WSA to obesity by Bagnall et al. [16]; Safefood [12] and Bleich et al [28]are included in Table 3. Examples of both process and outcome-based evaluations have been reported. Bagnall et al. [16] present their process analysis synthesis across all included studies making it difficult to ascertain evidence that relates specifically to obesity. Based on their summary table however it is likely that the process analysis for obesity studies is based on eight mixed methods evaluations [33, 34, 50, 54, 64–67] and seven qualitative studies [68–74]. Quality assessment of these studies is summarised in terms of numbers of studies meeting or not meeting quality criteria, but specific studies are not referenced in the text description covering this assessment. Many studies did not meet several quality criteria or did not provide sufficient information for an assessment to be made. The Safefood review [12] and the Bleich et al review [28] focused only on studies reporting quantitative outcome data. Although Bleich et al [28] engaged in risk of bias assessment and strength of evidence assessment for their nine included studies, they did not report this information by study. Instead, it was reported as an aggregate by setting of the intervention. All studies had either a high (n = 4) or moderate risk of bias (n = 5). Most studies were identified as providing insufficient strength of evidence (n = 6). Three studies based in both community and school settings were identified as providing moderate strength of evidence.

Outcome evaluations methods applied across these three reviews include: Two cluster randomised controlled trials [Sallis et al [61] (included in Bleich et al [28] only; quality rating not aligned with paper)]; Singh et al [53] (included in Bleich et al only; quality rating not aligned with paper)].

Seven non-randomised controlled trials [Johnson et al. [36] (included in Bagnall et al. [16] only; rated as good quality]; Sanigorski et al. [37] (included in Bagnall et al. [16] only; rated as good quality); Economos et al. [38] (included in all three reviews; rated as moderate to good quality by Bagnall et al. [16] and moderate quality bordering on strong by Safefood, [12]); Hoelsher et al. [60] (included in Bagnall et al. [16] only; rated as moderate quality); Bell et al. [49] (included in Safefood [12] only; rated as moderate quality); Verbestel et al. [51] (included in Safefood [12] only; rated as strong); Five mixed methods evaluations [DH, [54] (included in Bagnall et al. [16] only; rated as moderate quality); de Groot et al. [34] (included in Bagnall et al. [16] only; rated as moderate to poor quality); de Silva-Sanigorski et al. [35] (included in Bagnall and Safefood only; rated as moderate to poor quality by Bagnall et al. [16] and moderate quality by Safefood [12]); Amed et al. [50] (included in Bagnall et al. [16] only; rated as poor quality); Gadsby et al. [56] (included in Safefood [12] only; rated as poor quality); Two prospective cohort studies (Chomitz et al. [39] (Bagnall and Safefood only; rated as moderate quality by Bagnall et al. [16] and strong by Safefood [12]); Vinck et al. [58] (Safefood [12] only; rated as moderate quality bordering on strong); A prospective cohort study with embedded non-randomized controlled trial (de Henauw et al. [48] (included in Safefood [12] only; rated as moderate quality bordering on strong); Two pre-post designs [Atalla et al. [59] (included in Safefood [12] only; rated as moderate quality); Mead et al. [75] (included in Safefood [12] only; rated as moderate quality); One pre-post design with matched comparators from existing national survey data (Raine et al. [57] (included in Safefood [12] only; rated as moderate quality bordering on strong); an evaluation of unclear design [Schwarte et al. [33] (included in Bagnall et al. [16] only; unclear quality); And one nested case control study [Copeland et al. [55] (included in both Bagnall and Safefood reviews; rated as moderate to poor quality by Bagnall et al. [16] and as low quality by Safefood [12].

The Safefood Review [12] also included two articles focussed on the Amsterdam Healthy Weight Approach [40, 76] and presents data on changes seen at a whole-city level on rates of obesity that present reasons to be optimistic. Also, in a recent article by UNICEF [77] the positive outcomes of the AHWA are described. Full peer-reviewed publication of findings are still to emerge however, and until they are, attributing causality to the programme is cautioned against.

### RQ3. What evidence is there of the effectiveness of WSAs to diet, healthy weight, and obesity?

Only four articles evaluating the implementation of WSAs to diet, healthy weight and obesity were identified as being of good quality across the Safefood [12] and Bagnall et al. [16]reviews. All four of these articles report a positive effect on outcome variables related to healthy weight, diet, and obesity. Chomitz et al. [39], who report on the ‘Healthy Living Cambridge’ WSA, is reported to have found increases in physical fitness and decreases in BMI and BMI z score by Safefood and to have had positive effects on fitness and obesity by Bagnall. There are apparently conflicting reports about the direction of outcomes by Safefood and Bagnall on the impact of the ‘Be Active Eat Well’ WSA reported in Sanigorski et al. [37]. Safefood report *increased* BMI z scores whilst Bagnall et al. report positive effects on BMI and health behaviour. Closer inspection of the actual article reveals that children in the intervention arm gained less weight, had lower increases in waist circumference and lower increases in BMI z score and waist to height ratio than did controls but that there was no overall significant difference between groups in the incidence of overweight or obesity [37]. Bleich et al [28] reports a desirable effect of the WSA on BMI and BMI z score from baseline. The Johnson et al. paper [36] which was only included in the Bagnall review also reported on the ‘Be Active Eat Well’ WSA. Positive effects on BMI and health behaviour were identified. The ‘Shape Up Somerville’ WSA reported on by Economos et al. [38] and included in all three of the reviews was identified as having had a positive effect on (i.e., decrease in) BMI for participants. All four of these WSAs were identified as including at least some of the NICE guidance features for a WSA (at least 4/10 in the case of ‘Healthy Living Cambridge Kids’ (Chomitz et al., [39]) as reported by Bagnall et al. [16]. In addition, they were reported as having been developed by drawing on either socioecological theory or by using some kind of capacity building or community participatory approach.

Ten articles across the Bagnall et al and Safefood reviews were identified as being of moderate quality. The best ratings for studies included in the Bleich et al [28] review was moderate risk of bias and moderate strength of evidence, so we include commentary on the three WSA studies included only in their review here too [53, 61, 78]. These included two articles focused on the European Commission funded ‘IDEFICS’ WSA (De Henauw et al. [48]; Verbestel et al. [51]), two articles focussed on the ‘Romp and Chomp’ WSA {De Silva-Sanigorski et al. [35b]; De Groot et al. [34]), and two articles focussed on the UK ‘Change4Life’ WSA (Copeland et al. [55]; Department of Health, [54]). These thirteen studies identified mixed results on outcomes relevant to obesity, diet, and healthy weight. A number showed mainly negative or non-significant findings. The two ‘IDEFICS’ papers report increases in BMI z scores, fatness, body fat mass and waist to hip ratio (though it should be noted the target population were children who are still growing) (De Henauw et al. [48]) and decreases in physical activity and increases in sedentary time (Verbestel et al. [51]). In the ‘OPAL’ WSA, it was reported that there were no significant changes over time on multiple anthropometric measurements or BMI z score, multiple health behaviours including fruit and vegetable intake, nor health related quality of life (Bell et al. [49]). The ‘Change4Life’ WSA was reported to have had mixed effects on health and wellbeing outcomes with no reduction in obesity prevalence (Copeland et al. [55]), but positive effects on parental health behaviour and awareness (Department of Health [54]). Raine et al. [57] reported no significant changes in chronic disease risk, BMI, or blood pressure, Vinck et al. [58] reported no significant change in BMI. Chang et al [78] reported no significant change in prevalence of obesity and Singh et al [53] reported no significant differences between groups on change in BMI.

Two of the ten articles reported mixed findings within their data. Hoelshcer et al. [60] reported mixed effects for obesity prevalence and Mead et al. [75] reported increases in health knowledge and behaviour and healthy intentions, and decreases in unhealthy food consumption but no significant change to BMI was reported. The study by Sallis et al [61] evaluating the M-SPAN WSA, showed a desirable intervention effect for change in BMI, but only in boys. The one WSA reported on by moderate quality studies that had fully positive results was the ‘Romp and Chomp’ WSA. De Silva-Sanigorski et al. [35] report that it increased vegetable servings per day, increased physical activity and led to decreases in BMI in 3.5-year-olds. Parental awareness and community capacity building were also identified as positive outcomes in addition to positive effects on BMI by Groot et al. [34]. The WSA showing the most positive effects from these moderate quality studies (the ‘Romp and Chomp’ WSA) was not reported to have been developed or implemented using a theory, framework, or model. However, Bagnall et al. [16] identified the ‘Romp and Chomp’ programme as including 9/10 of the WSA NICE guidance features.

Four studies identified to be of poorer quality also report varied results. The Safefood review [12] reported on Atalla et al. [59] which identified increases in physical activity, increases in recommended fruit and vegetable intake and decrease in BMI among those with overweight or obesity. They also report on a study by Gadbsy et al. [56] with unclear reporting but including a non-significant decrease in BMI. Bagnall et al. [16] reported that the LIVE 5-2-1-0 WSA showed early indications of improvement in community awareness and action to promote healthy behaviours, but this data were not from robust outcome indicators. They also report on the ‘Central California Regional Obesity Prevention Program’ (CCROPP), which showed positive effects on nutrition and physical activity environments.

The Safefood review [12] also highlighted work on the Amsterdam Healthy Weight programme [40, 76] that was showing some promising early indicators of success, but an outcome evaluation was yet to be published at the time of the review, so caution was suggested in identifying this as an effective WSA at that time. Taken together the evidence for the effectiveness of WSAs in tackling population level overweight and obesity remains limited. Notably, the strongest study designs tend to suggest that WSAs are effective. However, more robust evidence with data captured over longer timeframes is still needed. The data also appear to suggest that adherence to all ten WSA NICE guidance features is not necessary for effective WSA development and impact, but may be helpful. So far there is no particular evidence that use of a specific guiding model, theory or framework is needed to support development of an effective WSA. It is noted however that participatory and capacity building approaches have been cited by several of the WSA studies where positive outcomes were achieved and we address the question of public involvement in WSA development and implementation next.

### RQ4. What has been the contribution of the public and/or service users in the development of WSAs?

The contribution of public and service user involvement in the development of WSA approaches has not been reported widely in the eight articles included in our review of reviews. This is with the exception of lived experience of food environments being identified in a systems map in the Sawyer et al. [27] review article and the importance of community involvement being highlighted in Bagnall et al. [16]. Consideration however of the primary studies that were included across the Bagnall et al. review [16] and the Safefood review [12] suggest that public involvement has played a significant role in the development, implementation, and evaluation of WSAs to diet, healthy weight, and obesity (see Table 3 above). For example, the four highest quality studies with positive outcomes reported across these two reviews explicitly state having either used a community-based participatory research approach (Chomitz et al., [39] ‘Shape Up Somerville’; Economos et al., [38] ‘Healthy Living Cambridge’) or took a capacity building approach, whereby communities were supported to create their own mix of solutions and interventions (Sanigorski et al. [37]; Johnson et al. [36]. Other poorer quality studies have also included WSAs that were developed with extensive community consultation and stakeholder engagement (e.g., De Silva-Sanigorski et al. [35]; De Groot et al. [34]; ‘Romp and Chomp’ which reported positive effects). Whilst public, end-user and stakeholder engagement cannot guarantee the success of a WSA for tackling diet, healthy weight and obesity, the evidence from these reviews suggests that it is an important component of getting the approach accepted by communities in which change is to take place [25].

## Discussion

Despite the increased application of WSAs to diet, healthy weight and obesity, evidence for effectiveness at a process or health outcome level of analysis remains in its infancy. The aim of this scoping review of reviews was to synthesize the evidence from reviews that investigated how whole systems approaches (WSA) to diet and healthy weight have been implemented and evaluated, what models or theories or frameworks have been used, what evidence there is of effectiveness and whether members of the public or service users have been involved in the process of developing, implementing and evaluating a WSA.

Collectively, several models, theories, frameworks, and key features of WSAs addressing diet, healthy weight and obesity have been described across the identified reviews and primary studies included in Safefood [12], Bagnall et al. [16] and Bleich et al [28] and were set out above and summarised within Tables 2 and 3. What is clear is that the range of frameworks, theories and models used in the development and implementation of the WSAs differs considerably from those identified at the review level to help describe or assess the evidence. For example, those who have developed WSAs have described drawing on socioecological theory, participatory research models, and models such as EPODE, the Re-Aim framework, Intervention Mapping, and an adapted Dahlgren & Whitehead Rainbow model, amongst others. Reviews within this field have used theories, models and frameworks in a number of different ways that include assessing the evidence against a framework (e.g., Johnston et al. [31]; Bagnall et al. [16]) or proposing a new systems model or framework or systems map to be used to develop WSAs in the future (e.g., Sawyer et al. [41]; Skinner & Foster [30], Nader et al [32]). One of the theories, models, or frameworks we thought we might encounter during the review process was evidence relating to what is commonly known as the Leeds Beckett Model (LBM) that was developed under commission from former Public Health England to support development and implementation of WSAs to tackle obesity in a United Kingdom context [11]. However, none of the reviews identified included reference to this. Evidence from evaluations of WSAs that have applied this approach are therefore needed as the LBM has been implemented in England, Scotland, Wales, and Northern Ireland without peer reviewed testing or independent evaluation [14]. Promising however, we are aware that some evaluations are underway [17].

Taken together, the evidence from this review does not suggest any one theory, framework, model, or approach might be favourable to others in development of an effective WSA. Instead, the evidence suggests that selecting one, especially one used in previously successful WSAs, may be helpful. Based on our assessment of the available models, if the aim is to map and understand obesity in a particular context or locality, systems dynamic models consisting of causal loop diagrams as described by Sawyer et al. [27] may be most appropriate. Developing these requires the input of systems scientists familiar with methods and software for developing casual loop diagrams and complex systems dynamics models and likely requires partnership working between academia and public health decision-makers and practitioners as well as other important stakeholders. If the aim is to integrate the systems map with policy interventions and community interventions with the target population being children, then the models used in the Childhood Obesity Modelling for Prevention and Community Transformation (COMPACT) study [29] may be relevant, as these take into account linking outcome assessments to changes to the obesogenic system.

Although the evidence on WSAs for tackling diet, healthy weight and obesity is still limited, there is reason to be optimistic about the potential of WSAs for offering a genuinely fruitful route to reversing or at least slowing the trend for increased levels of overweight and obesity at a population level. As our review shows, currently, only a handful of good quality studies exist that have evaluated WSAs. This evidence is drawn from non-RCTs and mixed methods studies, and typically identifies improvements on robust indicators of population level overweight and obesity (such as BMI z scores). Future research needs to continue to apply these higher quality designs and include longitudinal follow-up across included populations and their comparators. Many study descriptions of what constitute a WSA and the outcomes reported were limited, or lacked longitudinal follow-up. In particular, it was highlighted by Bagnall et al. [16] that very few of the reviewed articles reported on research where they set out to actually deliver a WSA, and therefore did not approach their evaluation from a systems perspective. Some of the newer frameworks (e.g., ENCOMPASS, LBM) that have been proposed could be considered more suitable for future implementation and evaluations. Furthermore, consistency in definition, application, and thorough evaluation of WSAs was lacking at the time of the reviews. With these research limitations highlighted, it makes it challenging to determine the effectiveness of WSAs to obesity, so any subsequent attempt to evaluate WSA could consider these shortcomings.

Our review has also identified that some of the most promising WSAs to date have had a strong stakeholder engagement and public involvement approach embedded from the outset. They have made use of participatory research approaches and focussed on capacity building so that interventions and solutions are designed by and with community members to suit and address local need. There has been relatively little focus on this issue within other reviews to date, making this review the first to draw attention to this aspect of WSA development. Arguably, given growing awareness of an appetite for co-design in the public health sector [25] further support, funding and advocacy for service user and public involvement is required in future development and implementation of WSA to obesity [14].

## Limitations

Like all research, this study is subject to some limitations. First, our search strategy was designed to look as widely as possible to minimise the risks of missing valuable research review studies however systems dynamics type studies may have been reported in computer science databases that we did not include. Second, our scoping review of reviews was restricted to only include reviews published in the English language, which may mean some useful evidence has been missed. It was also apparent when selecting reviews that a wide variety of names and definitions of WSAs were used, meaning it is possible we have missed reviews including relevant studies.

## Conclusion

WSA to diet, healthy weight and obesity show promise, yet the research evidence for their effectiveness is lagging behind implementation, with the exception of a few well-designed studies. Further robust evidence for WSAs to obesity are required that incorporate process and outcome evaluations, and that follow key guidance as described by NICE and Garside et al. [15] or Sawyer et al. [27] or for those who have used systems science models like Systems Dynamic Modelling. From the reviews included, it was apparent that a wide range of definitions were used to describe WSAs, we propose given the recent emphasis on whole systems implementation and training that international consensus is agreed on what constitute a WSA to obesity. This may require establishing an expert representative group to review definitions and come to agreement. Having a definition would then make future evaluation comparisons possible. It was not possible to determine how much funding can impact on the success of a WSA to obesity, this should be considered in the future. Furthermore, greater inclusion of members of the public, who are the ultimate target beneficiary of WSAs to obesity, in the development and implementation of WSAs should be considered. Research seeking to evaluate the impacts of WSAs should also do more to include and report upon the inclusion of the public and/or service users in their work. The use of existing epidemiological data alongside longitudinal process and outcome evaluation data to monitor the design, implementation, and long-term impact of WSAs to obesity should also be an ambition of future research. Finally, the role of psychology in understanding how public health groups are formed to initially steer whole systems approaches has been given little consideration. Although WSAs have been described with ‘the system’ and its functional components in mind, it is the people at the centre of that system that inevitable contribute to making it work, future work should consider how best to support these people in their roles especially given the limited guidance available. Our intentions is that this review of reviews will contribute to providing this much needed support.

## Supporting information

S1 File Screening tool for independent author screening. (XLSX)

S2 File Database search terms. (DOCX)

S1 Checklist PRISMA review checklist. (PDF)
