# Supplementary material for: Whole systems approaches to diet and healthy weight: A scoping review of reviews
Source: PLoS One. 2024 Mar 13;19(3):e0292945. doi: 10.1371/journal.pone.0292945 (PMC10936799; doi:10.1371/journal.pone.0292945)
Supplement: S2 File — (DOCX) [file pone.0292945.s002.docx]

**Search terms used for each database**

| **Search no.** | **Date searched** | **Database** | **Search terms** | **Limiters applied** | **Results** |
| --- | --- | --- | --- | --- | --- |
| 1 | 04/03/2022 | Medline | ‘diet’ OR ‘nutrition’ OR ‘eating habit’ OR ‘eating behaviour’ OR ‘food choice’ | None | 1,023,612 |
| 2 | 04/03/2022 | Medline | Whole system approach | None | 517 |
|  |  |  | 1 AND 2 | - English language - 1995-2021 - Review - Systematic review | 9 |
| 3 | 04/03/2022 | Medline | ‘diet’ OR ‘nutrition’ OR ‘eating habit’ OR ‘eating behaviour’ OR ‘food choice’ | None | 1,026,664 |
| 4 | 04/03/2022 | Medline | Systems approach | None | 44,398 |
|  |  |  | 3 AND 4 | - English language - 1995-2021 - Review - Systematic review | 363 |
| 5 | 04/03/2022 | Medline | ‘diet’ OR ‘nutrition’ OR ‘eating habit’ OR ‘eating behaviour’ OR ‘food choice’ | None | 1,023,612 |
| 6 | 04/03/2022 | Medline | System modeling | None | 13,801 |
|  |  |  | 5 AND 6 | - English language - 1995-2021 - Review - Systematic review | 41 |
| 7 | 21/02/2022 | Medline | ‘diet’ OR ‘nutrition’ OR ‘eating habit’ OR ‘eating behaviour’ OR ‘food choice’ | None | 1,023,612 |
| 8 | 21/02/2022 | Medline | ‘Interagency working’ OR ‘interagency collaboration’ OR ‘multiagency’ OR ‘multiagency working’ | None | 704 |
|  |  |  | 7 AND 8 | - English language - 1995-2021 - Review - Systematic review | 6 |
| 9 | 21/02/2022 | Medline | ‘diet’ OR ‘nutrition’ OR ‘eating habit’ OR ‘eating behaviour’ OR ‘food choice’ | None | 1,023,612 |
| 10 | 21/02/2022 | Medline | ‘partnership working’ OR ‘community-wide’ | None | 3,780 |
|  |  |  | 9 AND 10 | - English language - 1995-2021 - Review - Systematic review | 33 |
| 11 | 21/02/2022 | Medline | ‘obesity’ OR ‘obese’ OR ‘overweight’ OR ‘unhealthy weight’ OR ‘high bmi’ OR ‘body weight’ | None | 760,092 |
| 12 | 21/02/2022 | Medline | ‘whole system approach’ OR ‘interagency’ OR ‘interagency collaboration’ OR ‘interagency working’ OR ‘multiagency’ OR ‘multiagency working’ | None | 3,496 |
|  |  |  | 11 AND 12 | - English language - 1995-2021 | 10 |
| 13 | 04/03/2022 | CINAHL | ‘diet’ OR ‘nutrition’ OR ‘eating habit’ OR ‘eating behaviour’ OR ‘food choice’ | None | 275,354 |
| 14 | 21/02/2022 | CINAHL | Whole system approach | None | 290 |
|  |  |  | 13 AND 14 | - English language - 1995-2021 - Review - Systematic review | 3 |
| 15 | 04/03/2022 | CINAHL | ‘diet’ OR ‘nutrition’ OR ‘eating habit’ OR ‘eating behaviour’ OR ‘food choice’ | None | 275,354 |
| 16 | 21/02/2022 | CINAHL | Systems approach | None | 9,741 |
|  |  |  | 15 AND 16 | - English language - 1995-2021 - Review - Systematic review | 64 |
| 17 | 04/03/2022 | CINAHL | ‘diet’ OR ‘nutrition’ OR ‘eating habit’ OR ‘eating behaviour’ OR ‘food choice’ | None | 275,354 |
| 18 | 04/03/2022 | CINAHL | System modeling | None | 956 |
|  |  |  | 17 AND 18 | - English language - 1995-2021 - Review - Systematic review | 3 |
| 19 | 04/03/2022 | CINAHL | ‘diet’ OR ‘nutrition’ OR ‘eating habit’ OR ‘eating behaviour’ OR ‘food choice’ | None | 275,354 |
| 20 | 04/03/2022 | CINAHL | ‘Interagency working’ OR ‘interagency collaboration’ OR ‘multiagency’ OR ‘multiagency working’ | None | 551 |
|  |  |  | 19 AND 20 | - English language - 1995-2021 - Review - Systematic review | 0 |
| 21 | 04/03/2022 | CINAHL | ‘diet’ OR ‘nutrition’ OR ‘eating habit’ OR ‘eating behaviour’ OR ‘food choice’ | None | 275,600 |
| 22 | 04/03/2022 | CINAHL | ‘partnership working’ OR ‘community-wide’ | None | 2,470 |
|  |  |  | 21 AND 22 | - English language - 1995-2021 - Review - Systematic review | 13 |
| 23 | 04/03/2022 | CINAHL | ‘obesity’ OR ‘obese’ OR ‘overweight’ OR ‘unhealthy weight’ OR ‘high bmi’ OR ‘body weight’ | None | 233,599 |
| 24 | 04/03/2022 | CINAHL | ‘whole system approach’ OR ‘interagency’ OR ‘interagency collaboration’ OR ‘interagency working’ OR ‘multiagency’ OR ‘multiagency working’ | None | 1,782 |
|  |  |  | 23 AND 24 | - English language - 1995-2021 - Review - Systematic review | 6 |
| 25 | 04/03/2022 | Scopus | Diet OR nutrition OR “eating habit” OR “eating behaviour” OR “food choice” | None | 4,164,075 |
| 26 | 04/03/2022 | Scopus | “whole system approach” | None | 2,245 |
|  |  |  | 25 AND 26 | - Review - English language - 1995-2021 | 83 |
| 27 | 04/03/2022 | Scopus | Diet OR nutrition OR “eating habit” OR “eating behaviour” OR “food choice” | None | 4,164,075 |
| 28 | 04/03/2022 | Scopus | “Systems approach” | None | 224,505 |
|  |  |  | 27 AND 28 | - Review - English language - 1995-2021 | 2,782 |
| 29 | 04/03/2022 | Scopus | Diet OR nutrition OR “eating habit” OR “eating behaviour” OR “food choice” | None | 4,164,075 |
| 30 | 04/03/2022 | Scopus | “System modeling” | None | 172,464 |
|  |  |  | 29 AND 30 | - Review - English language - 1995-2021 | 499 |
| 31 | 04/03/2022 | Scopus | Diet OR nutrition OR “eating habit” OR “eating behaviour” OR “food choice” | None | 4,164,075 |
| 32 | 04/03/2022 | Scopus | ‘Interagency working’ OR ‘interagency collaboration’ OR ‘multiagency’ OR ‘multiagency working’ | None | 7,114 |
|  |  |  | 31 AND 32 | - Review - English language - 1995-2022 | 44 |
| 33 | 04/03/2022 | Scopus | Diet OR nutrition OR “eating habit” OR “eating behaviour” OR “food choice” | None | 4,164,075 |
| 34 | 04/03/2022 | Scopus | ‘partnership working’ OR ‘community wide’ | None | 32,303 |
|  |  |  | 33 AND 34 | - Review - English language - 1995-2022 | 885 |
| 35 | 23/02/2022 | Scopus | ‘obesity’ OR ‘obese’ OR ‘overweight’ OR ‘unhealthy weight’ OR ‘high bmi’ OR ‘body weight’ | None | 2,112,609 |
| 36 | 23/02/2022 | Scopus | ‘whole system approach’ OR ‘interagency collaboration’ OR ‘interagency working’ OR ‘multiagency’ OR ‘multiagency working’ | None | 9,336 |
|  |  |  | 35 AND 36 | - English language - 1995-2022 | 88 |
| 39 | 04/03/2022 | CINAHL | ‘diet’ OR ‘nutrition’ OR ‘eating habit’ OR ‘eating behaviour’ OR ‘food choice’ OR ‘food’ | None | 398,657 |
| 40 | 04/03/2022 | CINAHL | ‘whole system approach’ OR ‘systems approach’ OR ‘multiagency working’ | None | 9,773 |
| 41 | 04/03/2022 | CINAHL | Review | None | 691,959 |
|  |  |  | 39 AND 40 AND 41 | - English language - 1995-2021 | 79 |
| 42 | 04/03/2022 | CINAHL | ‘obesity’ OR ‘obese’ OR ‘overweight’ OR ‘unhealthy weight’ OR ‘unhealthy diet’ OR ‘fat’ OR ‘high bmi’ OR ‘high body mass index’ | None | 213,044 |
| 43 | 04/03/2022 | CINAHL | ‘whole system approach’ OR ‘interagency collaboration’ OR ‘interagency working’ OR ‘multiagency’ OR ‘multiagency working’ OR ‘partnership working’ OR ‘community-wide’ | None | 3,281 |
| 44 | 04/03/2022 | CINAHL | Review | None | 691,959 |
|  |  |  | 42 AND 43 AND 44 | - English language - 1995-2021 | 16 |
| 45 | 04/03/2022 | CINAHL | ‘obesity’ OR ‘obese’ OR ‘overweight’ OR ‘physical inactivity’ OR ‘physically inactive’ OR ‘physically active’ OR ‘unhealthy weight’ OR ‘healthy weight’ | None | 165,064 |
| 46 | 04/03/2022 | CINAHL | ‘whole system approach’ OR ‘systems approach’ OR ‘partnership working’ OR ‘collaborative working’ OR ‘multiagency’ OR ‘interprofessional collaboration' OR 'multiagency working' OR ‘interagency’ OR ‘joint working’ | None | 16,461 |
| 47 | 04/03/2022 | CINAHL | Review | None | 691,959 |
|  |  |  | 45 AND 46 AND 47 | - English language - 1995-2021 | 43 |
| 48 | 04/03/2022 | Medline | ‘diet’ OR ‘nutrition’ OR ‘eating habit’ OR ‘eating behaviour’ OR ‘food choice’ OR ‘food’ | None | 1,719,438 |
| 49 | 04/03/2022 | Medline | ‘whole system approach’ OR ‘systems approach’ OR ‘multiagency working’ | None | 44,320 |
| 50 | 04/03/2022 | Medline | Review | None | 2,566,022 |
|  |  |  | 48 AND 49 AND 50 | - English language - 1995-2021 | 492 |
| 51 | 04/03/2022 | Medline | ‘obesity’ OR ‘obese’ OR ‘overweight’ OR ‘unhealthy weight’ OR ‘unhealthy diet’ OR ‘fat’ OR ‘high bmi’ OR ‘high body mass index’ | None | 566,227 |
| 52 | 04/03/2022 | Medline | ‘whole system approach’ OR ‘interagency collaboration’ OR ‘interagency working’ OR ‘multiagency’ OR ‘multiagency working’ OR ‘partnership working’ OR ‘community-wide’ | None | 4,919 |
| 53 | 04/03/2022 | Medline | Review | None | 2,093,166 |
|  |  |  | 51 AND 52 AND 53 | - English language - 1995-2021 | 34 |
| 54 | 02/03/2022 | Medline | ‘obesity’ OR ‘obese’ OR ‘overweight’ OR ‘physical inactivity’ OR ‘physically inactive’ OR ‘physically active’ OR ‘unhealthy weight’ OR ‘healthy weight’ | None | 453,441 |
| 55 | 02/03/2022 | Medline | ‘whole system approach’ OR ‘systems approach’ OR ‘partnership working’ OR ‘collaborative working’ OR ‘multiagency’ OR ‘interprofessional collaboration' OR ‘interprofessional teamwork’ OR 'multiagency working' OR ‘interagency’ OR ‘joint working’ | None | 54,153 |
| 56 | 02/03/2022 | Medline | Review | None | 2,566,022 |
|  |  |  | 54 AND 55 AND 56 | - English language - 1995-2022 | 152 |
| 57 | 02/03/2022 | Scopus | ‘obesity’ OR ‘obese’ OR ‘overweight’ OR ‘unhealthy weight’ OR ‘unhealthy diet’ OR ‘fat’ OR ‘high bmi’ OR ‘high body mass index’ | None | 2,476,396 |
| 58 | 02/03/2022 | Scopus | ‘whole system approach’ OR ‘interagency collaboration’ OR ‘interagency working’ OR ‘multiagency’ OR ‘multiagency working’ OR ‘partnership working’ OR ‘community-wide’ | None | 41,475 |
| 59 | 02/03/2022 | Scopus | Review | None | 33,373,821 |
|  |  |  | 57 AND 58 AND 59 | - Review - English language | 678 |
| 60 | 02/03/2022 | Scopus | ‘obesity’ OR ‘obese’ OR ‘overweight’ OR ‘physical inactivity’ OR ‘physically inactive’ OR ‘physically active’ OR ‘unhealthy weight’ OR ‘healthy weight’ | None | 1,620,833 |
| 61 | 02/03/2022 | Scopus | ‘whole system approach’ OR ‘systems approach’ OR ‘partnership working’ OR ‘collaborative working’ OR ‘multiagency’ OR ‘interprofessional collaboration' OR ‘interprofessional teamwork’ OR 'multiagency working' OR ‘interagency’ OR ‘joint working’ | None | 296,966 |
| 62 | 02/03/2022 | Scopus | Review | None | 33,373,821 |
|  |  |  | 60 AND 61 AND 62 | - Review - English language | 1,903 |
| 63 | 02/03/2022 | Cochrane Library | ‘obesity’ OR ‘obese’ OR ‘overweight’ OR ‘unhealthy weight’ OR ‘unhealthy diet’ OR ‘fat’ OR ‘high bmi’ OR ‘high body mass index’ | None | 368 |
| 64 | 02/03/2022 | Cochrane Library | ‘whole system approach’ OR ‘interagency collaboration’ OR ‘interagency working’ OR ‘multiagency’ OR ‘multiagency working’ OR ‘partnership working’ OR ‘community-wide’ | None | 2622 |
| 65 | 02/03/2022 | Cochrane Library | Review |  | 1,881,507 |
|  |  |  | 63 AND 64 AND 65 | 01/01/1995 – 31/12/2021 | 104 |
| 66 | 02/03/2022 | Cochrane Library | ‘obesity’ OR ‘obese’ OR ‘overweight’ OR ‘physical inactivity’ OR ‘physically inactive’ OR ‘physically active’ OR ‘unhealthy weight’ OR ‘healthy weight’ | None | 71,572 |
| 67 | 02/03/2022 | Cochrane Library | ‘whole system approach’ OR ‘systems approach’ OR ‘partnership working’ OR ‘collaborative working’ OR ‘multiagency’ OR ‘interprofessional collaboration' OR ‘interprofessional teamwork’ OR 'multiagency working' OR ‘interagency’ OR ‘joint working’ | None | 8,307 |
| 68 | 02/03/2022 | Cochrane Library | Review | None | 1,881,507 |
|  |  |  | 66 AND 67 AND 68 | 01/01/1995 – 31/12/2021 | 1052 |
| 69 | 02/03/2022 | Cochrane Library | ‘diet’ OR ‘nutrition’ OR ‘eating habit’ OR ‘eating behaviour’ OR ‘food choice’ | None | 112,111 |
| 70 | 02/03/2022 | Cochrane Library | 'whole system approach' OR 'partnership working' OR 'collaborative working' OR multiagency OR 'multiagency working' OR 'joint working' OR 'interprofessional teamwork' OR 'interprofessional collaboration' | None | 4243 |
|  |  | Cochrane Library | Review | None | 1,881,511 |
|  |  |  | 69 AND 70 | 01/01/1995 – 31/12/2021 | 809 |
| 71 | 02/03/2022 | Cochrane Library | diet OR obesity OR 'healthy weight' OR 'unhealthy weight' OR 'eating habit' OR 'eating behaviour' OR 'body weight' OR fat OR 'high body mass index' OR bmi OR 'food choice' | None | 185,679 |
| 72 | 02/03/2022 | Cochrane Library | 'whole system approach' OR 'partnership working' OR 'collaborative working' OR multiagency OR 'multiagency working' OR 'joint working' OR 'interprofessional teamwork' OR 'interprofessional collaboration' OR 'systems approach' OR 'interagency working' | None | 8,307 |
|  |  | Cochrane Library | Review | None | 1,881,511 |
|  |  |  | 71 AND 72 | 01/01/1995 – 31/12/2021 | 2070 |
| 73 | 02/03/2022 | Cochrane Library | diet OR nutrition OR malnutrition OR 'eating habit' OR 'eating behaviour' OR 'food choice' OR 'unhealthy diet' OR 'healthy weight' OR 'unhealthy weight' OR obesity OR obese OR overweight OR underweight OR fat 'body fat' OR 'body mass index' OR 'body weight' OR 'physically inactive' OR 'physically active' OR 'physical inactivity' | None | 210395 |
| 74 | 02/03/2022 | Cochrane Library | 'whole system approach' OR 'partnership working' OR 'collaborative working' OR multiagency OR 'multiagency working' OR 'joint working' OR 'interprofessional teamwork' OR 'interprofessional collaboration' OR 'systems approach' OR 'interagency working' OR 'multi disciplinary' OR 'systems-based approach' 'cross-sector approach' OR 'multi-strategy approach' | None | 9244 |
|  |  | Cochrane Library | Review | None | 1,881,511 |
|  |  |  | 73 and 74 | 01/01/1995 – 31/12/2021 | 2230 |
